# Nicotinic acetylcholine receptor CHRNA5 is overexpressed in head and neck squamous cell carcinoma patients with a recent tobacco smoking history.

**DOI:** 10.17912/micropub.biology.001098

**Published:** 2024-02-02

**Authors:** Charlotte B McGuinness, Sara R White, Emma V Gray, Margaret V Leonard, Yong Teng, Austin Y Shull

**Affiliations:** 1 Department of Biology, Presbyterian College, Clinton, South Carolina, United States; 2 Department of Hematology and Medical Oncology, Winship Cancer Institute, Emory University, Atlanta, Georgia, United States

## Abstract

Tobacco smoking is a major driver of head and neck squamous cell carcinoma (HNSCC) occurrence, and previous studies have shed light on the precise molecular alterations in tobacco-related HNSCCs when compared to HNSCCs associated with other risk factors (ex: human papillomavirus/HPV status). In this study, we analyzed the gene expression differences in HNSCC cases with a recent smoking history and revealed that the nicotinic acetylcholine receptor CHRNA5 is differentially overexpressed in smoking-related HNSCCs. CHRNA5 overexpression in these HNSCCs corresponds with a worse prognosis and is inversely correlated with an immune expression signature commonly associated with better prognosis. From these results, our study highlights the potential role of the nicotine-activated CHRNA5 receptor in HNSCC progression and corresponds with other recent reports highlighting the potential role of nicotine induction in promoting cancer progression.

**Figure 1. Overexpression of nicotinic receptor CHRNA5 correlates with worse prognosis in HNSCC patients with a recent smoking history:**
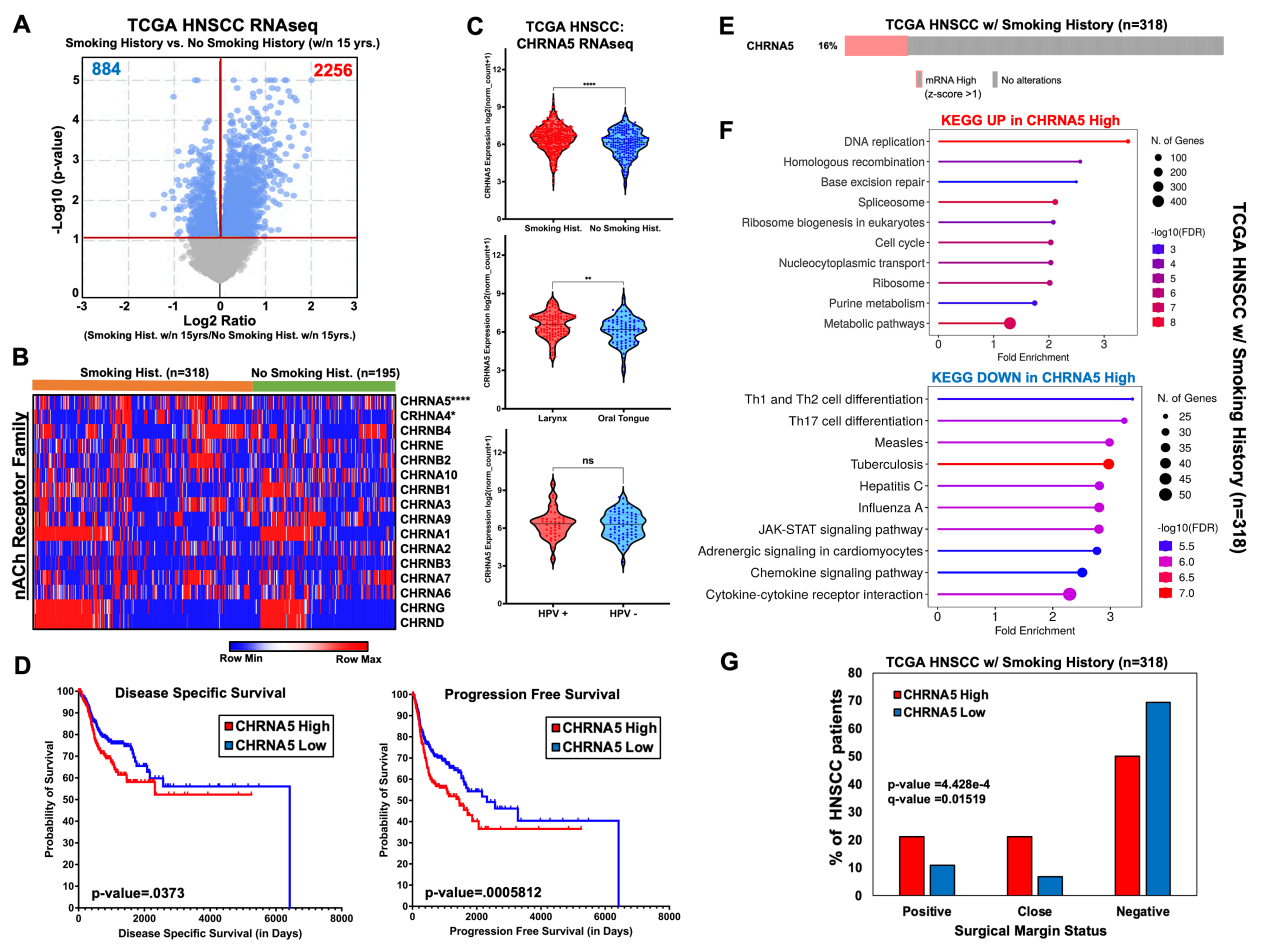
**Figure 1** **A)** Volcano plot demonstrating the differential gene expression differences (Student’s t-test, Benjamini-Hochberg corrected FDR *q* -value<0.05) between The Cancer Genome Atlas (TCGA) Head & Neck Squamous Cell Carcinoma (HNSCC) patients who either have a tobacco smoking history within the past 15 years or who do not have a tobacco smoking history within the past 15 years. **B)** Heatmap of TCGA HNSCC patient samples comparing the gene expression patterns of the nicotinic acetylcholine receptor (nAChR) family. **C)** Violin plots demonstrating CHRNA5 gene expression patterns based on smoking history vs. no smoking history, anatomic region of the diagnosed tumor (larynx vs. oral tongue), and human papilloma virus (HPV) status [Student’s t-test; ****p-value <0.0001, **p-value <0.01, ns=not significant]. **D)** Kaplan Meier (KM) survival plots showing a significant difference in disease specific survival and progression free survival based on ”high CHRNA5” or ”low CHRNA5” status. CHRNA5 status in TCGA HNSCC samples was determined using a CHRNA5 median expression threshold. **E)** OncoPrint plot showing the proportion of TCGA HNSCC patient samples with tobacco smoking history having a CHRNA5 gene expression z-score >1. **F)** KEGG Gene Ontology (GO) analysis for transcripts significantly upregulated in CHRNA5-high TCGA HNSCC samples with a smoking history as it compared to transcripts significantly downregulated in CHRNA5-high TCGA HNSCC samples with a tobacco smoking history. **G)** Histogram showing differences in surgical margin status between CHRNA5-high and CHRNA5-low TCGA HNSCC patients with a tobacco smoking history (Chi-squared test, Benjamini-Hochberg corrected).

## Description

Tobacco smoking is the leading risk factor in head and neck squamous cell carcinoma (HNSCC) development, with the second leading risk factor being human papilloma virus (HPV) infection. These risk factor subtypes differ greatly from both a clinical and molecular standpoint for this heterogenous cancer group that affects the oral cavity, pharynx, or larynx, with HPV-positivity generally exhibiting a better response to treatment and having a more favorable overall prognosis when compared to their tobacco smoking-related HNSCC counterparts. This dichotomy in outcomes underscores the need for defining the precise molecular mechanisms that differentially drive these different HNSCC subtypes, especially with the specific context that smokers with HNSCC often experience more aggressive disease progression, higher rates of recurrence, and diminished response to treatment regimens (Johnson et al. 2020) .

Because of these clinical hurdles related to tobacco-smoking associated HNSCC cases, we sought to better characterize the transcriptional landscape of HNSCC patients who have a recent history with tobacco smoking compared to those who have no smoking history within a 15-year time frame of diagnosis (fifteen years of tobacco cessation is often used as a time frame for typical risk factors to become normalized with individuals who have no smoking history) (U.S. Department of Health and Human Services 2020) . For this approach, we performed differential gene expression analysis using The Cancer Genome Atlas (TCGA) HNSCC dataset of 513 HNSCC patient samples with an identified smoking history (Leemans et al. 2018) and determined that 2256 transcripts were differentially upregulated and 884 transcripts downregulated in HNSCC patient samples with a recent smoking history. Such transcripts upregulated included oxidative stress response genes like *UGT1A1* [Fold Change: 1.72, FDR p-value = 5.095e-5] (Yueh and Tukey 2007) , *CYP1A1* [Fold Change: 2.30, FDR p-value = 5.39e-8] (Coelho et al. 2022) *, FGF19* [Fold Change: 2.19, FDR p-value = 2.135e-5] (Teng et al. 2017; Gao et al. 2019) *,* and *AKR1C1* [Fold Change: 2.14, FDR p-value = 7.265e-5] (Chang et al. 2019) , whereas transcripts downregulated included inflammatory genes like *IL4R* [Fold Change: 0.78, FDR p-value = 8.902e-5] (Bankaitis and Fingleton 2015) , *MYD88* [Fold Change: 0.83, FDR p-value = 3.258e-4] (Geng et al. 2010) , *CASP1* [Fold Change: 0.74, FDR p-value = 2.868e-4] (Song et al. 2022) , and *STAT2* [Fold Change: 0.82, FDR p-value = 9.424e-4] (Yang et al. 2022) ( Figure 1A ). These differently expressed transcripts logically correspond with the dichotomy of molecular characteristics between stress-resistant tobacco-related cancers and immunologically hot HPV+ cancers (Johnson et al. 2020) .

Interestingly, two of the differentially upregulated transcripts in HNSCC patients with a smoking history are the nicotinic acetylcholine receptor subunits CHRNA5 [Fold Change: 1.31, FDR p-value = 1.905e-3] and CHRNA4 [Fold Change: 1.38, FDR p-value = .0112], with CHRNA5 being more significantly upregulated of the two transcripts based on a Benjamini-Hochberg corrected FDR p-value. These two subunits are two of the sixteen subunits that comprise the overall nicotinic receptor family in humans, where different heteropentameric structures make up the varying nicotinic receptor complexes throughout the body ( Figure 1B ) (Millar and Harkness 2008; Dang et al. 2016) . Interestingly, several studies have highlighted CHRNA5’s dynamic role in nicotine sensitivity as well as its potential oncogenic role in different cancer types (Improgo et al. 2010; Lin et al. 2019; Fu et al. 2022) . To further validate CHRNA5’s role with tobacco related HNSCC, we observed the CHRNA5 expression was significantly overexpressed in HNSCC tumors from the larynx when compared to the oral tongue, as most tobacco-related HNSCCs occur in the laryngeal region whereas oral tongue HNSCCs are more associated with HPV positivity (Johnson et al. 2020) . Furthermore, as expected, we observed no statistically significant difference in CHRNA5 expression between HPV+ and HPV- HNSCC tumors ( Figure 1C ). To further investigate CHRNA5’s connection with tobacco-related HNSCC, we analyzed the survival status of patients based on CHRNA5 expression and discovered that high CHRNA5 corresponded to a significantly lower disease-specific survival and significantly lower progression-free survival, both of which correspond with typical observation that tobacco-related HNSCCs have worse prognoses ( Figure 1D ).

From these initial results, we then isolated TCGA HNSCC samples that had a tobacco smoking history (n=318) to further focus on the differential CHRNA5 expression status within this subset of HNSCC patients and postulate on its specific contribution in smoking-related HNSCC cancers. From these 318 patients, sixteen percent of patients had a CHRNA5 expression z-score >1, which we then used as our cutoff in determining which tobacco associated HNSCC patients are considered “CHRNA5 High” ( Figure 1E ). We then compared the differentially expression of transcripts in “CHRNA5 High” HNSCC patients with a smoking history versus “CHRNA5 Low” HNSCC patients with a smoking history and observed that transcripts upregulated in CHRNA5 High HNSCC tumors were involved in DNA repair processes (ex: Base Excision Repair, Homologous Recombination, etc.) whereas transcripts downregulated in CHRNA5 High tumors were involved immune regulation pathways (Th1 and Th2 cell differentiation, JAK-STAT signaling pathway, etc.) ( Figure 1F ). This result provided evidence that CHRNA5 expression negatively corresponds to immune activation pathways, which is a negative correlation also generally observed in tobacco related HNSCC tumors. Furthermore, these gene ontology patterns observed in these HNSCC tumor subsets relate with the clinical observation that CHRNA5 High status in HNSCC patients with a Smoking History have a higher percentage of patients with “Positive” or “Close” surgical margins, which is an indicator of disease progression and worse prognosis ( Figure 1G ).

With the combined clinical and transcriptional data from TCGA concerning tobacco-related HNSCC patients, we observe that the nicotinic acetylcholine receptor subunit CHRNA5 corresponds with smoking history and may play both a prognostic and mechanistic role in HNSCC disease progression. These results concerning CHRNA5 are highly important as they correspond with other studies highlighting CHRNA5’s role in cancer progression in cancer types like lung and breast (Sun et al. 2017; Zhang et al. 2017; Koker et al. 2018) . Specifically, one study showing increased lung cancer cell aggressiveness due to nicotine treatment could be mitigated when knocking down CHRNA5 expression with siRNA. This result of a nicotine-induced CHRNA5 axis is especially interesting considering nicotine use in electronic cigarettes. Therefore, our analysis in HNSCC along with previous reports in other cancers associated with tobacco use highlight the potential importance of nicotinic receptor CHRNA5 in cancer progression and the potentially overlooked role nicotine induction via CHRNA5 may play in cancer severity.

## Methods

*Data Acquisition and RNAseq Differential Expression Analysis*

Clinical information and RSEM-normalized RNAseq expression counts from TCGA Head and Neck Squamous Cell Carcinoma (HNSCC) cohort were securely obtained using cBioPortal for Cancer Genomics platform (Gao et al. 2013; Gao et al. 2022) . TCGA HNSCC datasets used for this analysis were from the Firehose Legacy datasets maintained through the Broad Institute. Differential gene expression analysis and volcano plot construction was performed using the cBioPortal platform. A Benjamini-Hochberg FDR corrected p-value (i.e. q value) < 0.05 was set as the threshold for statistical significance for differential expression. Survival data for TCGA HNSSC samples were obtained using the UCSC Xena Browser platform (Goldman et al. 2019) . The OncoPrint in Figure 1E was generated in cBioPortal .

*Heatmap construction*

Heatmap of the normalized RNAseq expression counts for the nAChR gene family was constructed using the Morpheus matrix visualization and analysis software maintained by the Broad Institute (https://software.broadinstitute.org/morpheus). TCGA HNSCC samples were grouped based on tobacco smoking history status and were then clustered based on Euclidean distance to create the heatmap shown.

*Violin and bar plots*

Violin plots of the HNSCC subgroups for CHRNA5 expression were created using GraphPad Prism version 10. A Student’s t-test was used to determine statistical significance between the group comparisons (p-value < 0.05). Bar plot showing surgical margin status was constructed in Microsoft Excel 2021. A Chi-squared test was used to determine statistical significance of surgical margin status between the two CHRNA5 status groups.

*Survival analysis*

A Kaplan-Meir plot for disease-specific survival and progression-free survival was constructed using GraphPad Prism version 10. Grouping of CHRNA5 High and CHRNA5 Low for the KM survival analysis was determined using the median expression count value for all TCGA HNSCC patients with RNAseq information as the threshold separating the two groups. A log-rank test was used to determine statistical significance (p-value < 0.05).

*Gene Ontology (GO) analysis*

GO analysis of the Kyoto Encyclopedia of Gene and Genomes (KEGG) pathways was performed using the ShinyGO 0.77 platform (Ge et al. 2020) . Transcripts that were significantly upregulated and significantly downregulated in CHRNA5 high HNSCC tumors (cutoff FDR p-value<0.05) with a smoking history were used as inputs to perform the GO analysis. An FDR q-value of 0.05 was used as cutoff for significant pathways in the analysis, and the lollipop charts show the top 10 pathways ranked based on fold enrichment with size of the circle representing overall number of genes overlapping with KEGG pathway, and the color of each lollipop line representing the -log10 transformed FDR q-value.
